# Dynamic Changes of Treg and Th17 Cells and Related Cytokines Closely Correlate With the Virological and Biochemical Response in Chronic Hepatitis B Patients Undergoing Nucleos(t)ide Analogues Treatment

**DOI:** 10.5812/hepatmon.15332

**Published:** 2013-12-23

**Authors:** Xue-Ping Yu, Ru-Yi Guo, Mi-Long Su, De-Song Ming, Cheng-Zu Lin, Yong Deng, Zhen-Zhong Lin, Zhi-Jun Su

**Affiliations:** 1 Department of Infectious Diseases, the First Hospital of Quanzhou, Fujian Medical University, Quanzhou, China; 2 Clinical Laboratory, the First Hospital of Quanzhou, Fujian Medical University, Quanzhou, China; 3 Department of Infectious Diseases, the Second People's Hospital of Pingxiang, Pingxiang, China

**Keywords:** Hepatitis B, Chronic, T-Lymphocytes, Regulatory, Th17 Cells, Cytokines

## Abstract

**Background:**

The restoration of HBV-specific T-cell response during antiviral therapy is associated with CD4+T-cell activity. Treg cells and Th17 cells are subtypes of CD4+T cell. However, it has remained unknown how the Treg and Th17 cells and their associated cytokines affect nucleos(t)ide analogues (NA) antiviral efficacy.

**Objectives:**

The aim of the present study was to provide a new insight to evaluate the NA antiviral therapy for patients with chronic hepatitis B (CHB).

**Patients and Methods:**

Forty-four CHB patients hospitalized between July 2010 and August 2011 were enrolled in this study. They were received NA (entecavir, lamivudine and adefovir) treatment for 14.42 ± 13.08 weeks, and the peripheral blood was collected. The frequencies of Treg and Th17 cells were detected by flow cytometric analysis, and the levels of IL-10, TGF-β1, IL-17 and IL-23 were measured by enzyme-linked immunosorbent assay (ELISA).

**Results:**

In complete and partial-responders, Treg cells frequencies and IL-10, TGF-β1, IL-23 levels were all decreased significantly after NA therapy, while Th17 cells and the IL-17 levels were increased slightly. Treg/Th17 ratio was only dramatically declined in complete-responders. But there was no significant difference in non-responders. Either HBV DNA decreased by at least 2 log copies /mL or ALT turned to normal level, Treg cells frequencies and IL-10, TGF-β1, IL-23 levels were significantly reduced. Meanwhile, Treg cells were positively correlated with HBV DNA and ALT.

**Conclusions:**

The changes of Treg and Th17 cells and their associated cytokines were related to virological and biochemical responses.

## 1. Background

Hepatitis B virus (HBV) infection is a serious threat to human health. There are about two billion people, infected with HBV all over the world, and 360 million have chronic infection, and 600 thousand die annually from HBV-related liver disease (1). Over 10% of population in some Asian and Western Pacific countries have chronic HBV infection, and it continues to be highly variable (2). HBV itself does not cause liver disease, but the abnormal immune response between the host and virus in liver cells can affect the clinical outcome of HBV infection and clinical antiviral efficacy (3). Nucleoside (acid) analogues (NA) and interferon are approved antivirus therapies in the treatment of patients with chronic hepatitis B (CHB). NA (lamivudine, adefovir, entecavir, telbivudine, and tenofovir) can effectively inhibit HBV replication, and reduce liver inflammation and disease progression of cirrhosis (4). However, the sensitivity of the NA varies individually, resulting different antivirus effects (5, 6). Therefore, an early immunological parameter with close correlation to immune status is necessary to assess the NA antiviral efficacy. An immune disorder or imbalance exists in CHB patients. CD4 + T cell can modulate the host immune response by releasing special immune factors. Treg cells are differentiated from naive T-cell precursors, accounting for about 5-10% of the peripheral CD4 + T cells (7). Treg cells effectively inhibit other immune cells by secreting cytokines IL-10 and TGF-β1, to mediate the immune tolerance and maintain the immune balance (8, 9). Studies have shown that Treg cells can suppress HBV-specific immune responses, indirectly influencing the disease progression (10, 11). Th17 cells, a new discovery subtype of CD4 + T cell are characterized by IL-23 dependence and IL-17 secretion (main effect cytokine) (12-14). Many studies have shown that Th17 cells have important antimicrobial and antifungal effects in the host cell (15, 16), whereas the mechanism of anti-HBV is not yet clear enough.

Although Treg and Th17 cells are differentiated from a same precursor of CD4 + T cell, they are mutual restricted functionally and mutual transformed in differentiation and the balance of Treg/Th17 plays an important role in mediating the immune response of anti-HBV therapy (17, 18). Zhang et al. (13) indicated that during the first three months of entecavir treatment, Treg/Th17 ratio decreased correlatively, together with the inhibition of HBV DNA level. Zheng et al. (19) found that telbivudine treatment reduced HBV DNA level, as well as Treg/Th17 cells and related cytokines, while Th2 cells increased significantly. These findings indicate that after NA treatment, changes of Treg and Th17 cells are associated with HBV DNA replication level. However, the virological and biochemical response of NA treatment has not been reported. More importantly, telbivudine was improved to be immune activator, which can increase the CD4+ and CD8 + T cell responsiveness (20). Therefore, the observation of telbivudine treatment did not reflect the natural anti-HBV immunity after NA treatment.

## 2. Objectives

In this study, we performed a longitudinal investigation of the changes in the frequencies and related cytokines of Treg and Th17 cells before and after NA (lamivudine, adefovir, and entecavir, which were not proved to be immune activators) antivirus therapy. Furthermore, we discussed the association between these changes and virological and biochemical response, aiming to provide a new insight to evaluate the antiviral therapy for CHB patients.

## 3. Patients and Methods

### 3.1. Patients

Peripheral blood was collected from 44 CHB patients hospitalized from July 2010 to August 2011 at the Department of Infectious Diseases of the First Hospital of Quanzhou, affiliated with Fujian Medical University. There were 34 males and 10 females, 26 cases of hepatitis B e antigen (HBeAg)-positive CHB patients, and 18 cases of HBeAg-negative CHB patients, with an average age of 32.93±9.64 years old, ranging from 21 to 67 years old. CHB diagnostic criteria were described in detail previously (21). The screening criteria were as follows: (1) All patients with positive results for hepatitis B surface antigen (HBsAg) for more than 6 months, and HBV DNA of ≥ 105 copies/mL in HBeAg-positive CHB patients, or HBV DNA of ≥ 104 copies/mL in HBeAg-negative patients; (2) Alanine aminotransferase (ALT) level of ≥ 2 times the upper limit of normal; (3) Not received immunomodulating agents, such as thymosin and glucocorticoid hormones, or antivirus therapy; and (4) Those infected with hepatitis A virus (HAV), hepatitis C virus (HCV), hepatitis D virus (HDV) or human immunodeficiency virus (HIV), and those with alcohol- or drug-induced autoimmune liver diseases. 

Written informed consent was obtained from all patients, and the study protocol was approved by the Ethics Committee of the first hospital of Quanzhou affiliated to Fujian Medical University.

### 3.2. Treatment and Sample Collection

All patients signed a written informed consent for acceptance of NA treatment. Thirty patients received entecavir (Sino-American Shanghai Squibb Pharmaceuticals Ltd, 0.5 mg/d); 10 patients received lamivudine (GSK, Tianjin, China, 100 mg/d); and 8 patients received adefovir dipivoxil (TIPFR Pharmaceutical Responsible Co., Ltd., 10 mg/d). Among them, 4 patients received both lamivudine (100 mg/d) and adefovir dipivoxil (10 mg/d). Peripheral venous blood was collected before and 14.42 ± 13.08 weeks after NA antivirus therapy in two tubes respectively, one for detection of Treg cells and Th17 cells; and the other for measurement of cytokine IL-17, IL-23, IL-10 and TGF -β1 after centrifugation and freezing at -80 °C. 

### 3.3. Grouping According to Antiviral Efficacy

26 HBeAg- positive patients were divided into 3 groups according to antiviral efficacy (3) after taking NA for 14.42 ± 13.08 weeks. In complete-response group, HBeAg and HBV DNA converted to negative, and ALT normalized. In partial-response group, ALT was decreased or normalized, and HBV DNA converted to negative or decreased by at least 2 log copies/mL, and without HBeAg negative. In non-response group, none of the above indicators met the criteria. 

Eighteen HBeAg- negative patients were divided into 3 groups. In complete-response group, HBV DNA converted to negative and ALT normalized. In partial-response group, HBV DNA converted to negative or decreased by at least 2 log copies/mL, and ALT was decreased or normalized. In non-response group, none of the above indicators met the criteria.

### 3.4. Flow Cytometry Analysis

For analysis of Th17 cells, the cell suspension was stimulated with 25 ng/mL of phorbol 12-myristate-13-acetate (PMA) and 1 μg/mL ionomycin (Ion) in the presence of 1.7 μg/mL monensin (Mon) (eBioscience, San Diego, CA, The USA) in six-well plates. After 5 hours of culture at 37℃ with 5% CO2, the cells were transferred to tubes and washed once in PBS, then incubated with 10 μL PE-cy5-conjugated antihuman CD3 and 20 μL PE-conjugated antihuman CD8 (Beckman Coulter Immunotech, Webster, TX, The USA) at room temperature for 15 minutes. After surface staining, the cells were fixed and permeabilized according to the manufacturer's instruction, and then stained with FITC-conjugated antihuman IL-17A (eBioscience, San Diego, CA, The USA).

Recent studies suggested a combination of high expression of CD4 and CD25 and low expression of CD127 to identify Treg cells which have a high grade of inhibitory activity and high expression of FoxP3 (22). Therefore, we chose CD4 + CD25 high CD127 low T cells to determine the frequencies of Treg cells. To analysis Treg cells, the cells were incubated with 20 μL PE-conjugated antihuman CD127 (eBioscience, San Diego, CA, The USA), 20 μL FITC-conjugated antihuman CD4 (Beckman Coulter Immunotech, Webster, TX, The USA), and 10 μL PE-cy5-conjugated antihuman CD25 (Beckman Coulter Immunotech, Webster, TX, The USA) at room temperature for 20 minutes. All cells suspensions were treated with 2 mL erythrocyte lysis buffer (Invitrogen, Carlsbad, CA, The USA) for 20 minutes. Isotype controls were used to correct nonspecific binding. Th17 cells (CD3 + CD8-IL-17 + T cells) and Treg cells (CD4 + CD25highCD127low T cells) were analyzed by flow cytometry (Beckman Coulter Epics XL, Fullerton, CA, The USA).

### 3.5. Enzyme Linked Immunosorbent Assays (ELISA)

The concentrations of IL-17, IL-23, IL-10 and TGF-β1 in plasma were measured by ELISA kit (MARKET INC, SAN JOSE, CA, The USA) in accordance with the manufacturer's instructions. The data were read at 450 nm by a microplate reader (Alisei Quality System, SEAC, Italy).

### 3.6. Statistical Analysis

All data were analyzed with SPSS version 13.0 (SPSS Inc., Chicago, IL, The USA). Continuous variables expressed as mean ± standard deviation. The Mann-Whitney nonparametric U test was used for comparison between the two groups. The Wilcoxon signed-rank test was used for paired comparisons. The Spearman's rank correlation was performed between the variables. P value < 0.05 was considered statistically signiﬁcant.

## 4. Results

**4.1. Change in Treg Cells and Th17 Cells**,** and the Level of Their Related of Cytokines After Nucleos(t)**ide** Analogues Antivirus Therapy**

There were 14 complete -responders, 15 partial-responders, and 15 non-responders in 44 CHB patients after taking NA for 14.42 ± 13.08 weeks. Among the HBeAg- positive CHB patients, there were 6 complete-responders, 11 partial-responders and eight non-responders; among the HBeAg-negative CHB patients, there were eight complete- responders, 4 partial- responders, and 7 non- responders. 

**Figure 1.**
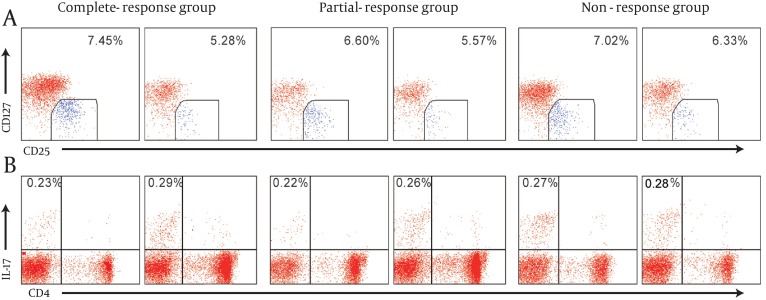
The Changes of Treg and Th17 Cells in Different Responders CHB Patients Before and After Nucleos(t)ide Analogues Antivirus Therapy A representative CD127 low expression in CD4 + CD25 high T subsets from each response group was shown. B representative IL-17A expression in CD3 + CD8 T subsets (CD4 + subsets) form each response group was shown. The mean percentage of positive cells was shown in each panel.

Before NA antivirus therapy, there were no significant differences among complete-response group, partial-response group, and no-response group in Treg cells and Th17 cells frequencies, Treg/Th17 ratio, and IL-10, TGF-β1, IL-17, and IL-23 levels. After NA treatment for 14.42 ± 13.08 weeks, in complete-response group, the levels of IL-10, TGF-β1 and IL-23 and Treg/Th17 ratio decreased significantly compared to the values before the treatment, and Th17 cells and IL-17 increased slightly with no significant difference. In partial-response group, Treg cells and levels of IL-10, TGF-β1 and IL-23 decreased significantly compared to the values before the treatment, and Th17 cells frequencies and IL-17 level increased and Treg/Th17 ratio decreased slightly. In non-response group, Treg cells, L-10, TGF-β1, IL-23, and Treg/Th17 ratio decreased slightly, Th17cells and IL-17 increased slightly, and there were no significant differences (Figure 1, and Table 1). 

**Table 1.** The Frequencies of Treg and Th17 Cells and the Levels of Treg/Th17 Cell-Associated Cytokines in Different Responders CHB Patients Before and After Nucleos(t)ide Analogues Antivirus Therapy ^a^

Group	n	Treg	Treg cell		Th17	Th17 cell		Treg/Th17 Ratio
			IL-10	TGF-β1		IL-17	IL-23	
**Complete responders**	14							
Before		7.45 ± 3.23	95.80 ± 73.60	106.18 ± 236.74	0.23 ± 0.19	102.73 ± 36.11	5296.03 ± 4323.81	54.47 ± 39.85
After		5.28 ± 1.23 ^b^	68.72 ± 65.33 ^b^	69.26 ± 152.924 ^b^	0.29 ± 0.27	122.60 ± 56.16	4000.31 ± 3541.20 ^b^	27.44 ± 15.22 ^b^
**Partial responders**	15							
Before		6.60 ± 1.61	106.76 ± 144.48	123.73 ± 65.69	0.23 ± 0.21	129.49 ± 54.55	7164.08 ± 8149.08	58.37 ± 59.99
After		5.57 ± 1.75 ^b^	72.20 ± 117.06 ^b^	34.33 ± 21.97 ^b^	0.26 ± 0.17	132.88 ± 47.47	4213.56 ± 5846.18 ^b^	32.85 ± 22.14
**No responders**	15							
Before		7.17 ± 1.96	101.19 ± 113.96	44.90 ± 72.47	0.28 ± 0.15	126.80 ± 46.18	5806.54 ± 6425.62	53.14 ± 91.02
After		6.33 ± 2.04	93.84 ± 101.76	35.17 ± 39.94	0.26 ± 0.19	142.30 ± 45.85	5561.56 ± 6750.20	39.44 ± 30.45

^b^ P < 0.05.

^a^Treg and Th17 cells frequencies are represented in percentage, the levels of cytokines are represented with pg/mL. Data are shown as mean ± standard deviation, and analyzed by Wilcoxon signed-rank test. The frequencies of Treg cells, the levels of IL-10, TGF-β1 and IL-23 were all decreased significantly in complete-responders and partial-responders, and Treg/Th17 ratio was only dramatically declined in complete-responders compared to the values before the antiviral therapy.

### 4.2. Association Between HBV DNA Decline and Treg Cells, Th17 Cells and Their Related Cytokines After Nucleos (t)ide Analogues Antivirus Therapy

After 14.42 ± 13.08 weeks of NA antivirus therapy, HBV DNA decreased by ≥ 2 log copies/mL in 19 patients, ≥ 1 log copies/mL in 11 patients, and < 1 log copies/mL in 14 patients. There were 11, 4, and 11 patients among the HBeAg- positive CHB group, and 8, 7, and 3 patients among the HBeAg- negative CHB group, accordingly. In patients with HBV DNA decline ≥ 2 log copies/mL, Treg cells frequencies and IL-10, TGF-β1, IL-23 levels decreased significantly, Th17 cells and IL-17 increased slightly, Treg/Th17 ratio decreased slightly compared to the values before therapy. In patients with HBV DNA decline ≥ 1 log copies/mL and <1 log copies/mL, Treg cells and cytokines IL-10, TGF-β1, IL-23, and Treg/Th17 ratio decreased slightly, Th17 cells and IL-17 increased slightly compared to the values before therapy (Table 2).

**Table 2.** The Frequencies of Treg and Th17 Cells and the Levels of Treg/Th17 Cell-Associated Cytokines in Different HBV DNA Decreased Groups (%, pg/mL, x ^-^ ± s) ^a^

HBV DNA log copies /mL[Table-fn fn6411]	n	Treg	Treg cell		Th17	Th17 cell		Treg/Th17 Ratio
			IL-10	TGF-β1		IL-17	IL-23	
**< 1**	14							
Before		7.05 ± 1.80	120.20 ± 127.48	24.06 ± 19.68	0.28 ± 0.18	130.42 ± 44.82	6091.57 ± 7231.64	61.87 ± 106.13
After		6.00 ± 2.01	105.13 ± 108.83	27.12 ± 36.54	0.25 ± 0.20	141.88 ± 49.44	6827.78 ± 7209.81	42.40 ± 34.27
**≥ 1**	11							
Before		6.52 ± 2.15	124.02 ± 147.37	96.51 ± 99.11	0.18 ± 0.09	118.55 ± 54.24	7085.46 ± 7841.36	50.77 ± 38.50
After		5.78 ± 1.97	100.84 ± 126.94	41.20 ± 36.25	0.27 ± 0.18	140.52 ± 47.89	6058.88 ± 6823.22	29.64 ± 17.90
**≥ 2**	19							
Before		7.56 ± 2.75	82.18 ± 76.50	118.33 ± 203.25	0.27 ± 0.20	108.62 ± 40.02	5235.15 ± 4989.70	44.42 ± 31.86
After		5.76 ± 1.77 ^b^	56.84 ± 62.68 ^b^	62.94 ± 130.54 ^b^	0.29 ± 0.24	123.94 ± 52.73	3312.48 ± 3178.43 ^b^	30.37 ± 18.85

^b^P < 0.05.

^a^Treg and Th17 cells frequencies are represented in percentage, the levels of cytokines are represented with pg/mL. Data are shown as mean ± standard deviation, and analyzed by Wilcoxon signed-rank test. The frequencies of Treg cells, the levels of IL-10, TGF-β1 and IL-23 were significantly reduced when HBV DNA decreased by at least 2 log copies /mL compared to the values before the antiviral therapy.

### 4.3. Association Between Treg Cells, Th17 Cells and Their Related Cytokines and Normal ALT After Nucleos(t)ide Analogues Antivirus Therapy

The ALT level decreased to less than 40 U/L in 24 patients (normal group) 14.42±13.08 weeks after NA antivirus therapy; while it was still above 40 U/L in 20 patients (abnormal group). Among the HBeAg- positive CHB patients, there were 13 patients whose ALT level decreased to less than 40U/L, and 13 patients whose ALT level was still above 40 U/L. Among the HBeAg- negative CHB patients; there were 11 patients whose ALT level decreased to less than 40U/L, and 7 patients whose ALT level was still above 40 U/L. 

In the ALT normal group, frequencies of Treg cells and levels of IL-10, TGF-β1 and IL-23 and Treg/Th17 ratio decreased significantly compared to the values before the treatment, and the frequencies of Th17 cells and IL-17 cells increased slightly. In the ALT abnormal group, there were no significant changes in frequencies of Treg cells and Th17 cells and levels of their related cytokines after the treatment (Table 3). 

**Table 3.** The Frequencies of Treg and Th17 Cells and the Levels of Treg/Th17 Cell-Associated Cytokines in ALT Normal and Abnormal Groups (%, pg/m, x ^-^ ± s) ^a^

Group[Table-fn fn6412]	n	Treg	Treg cell		Th17	Th17 cell		Treg/Th17 Ratio
			IL-10	TGF-β1		IL-17	IL-23	
**ALT Normal**	24							
Before		7.18 ± 2.72	92.22 ± 101.83	101.15 ± 187.33	0.24 ± 0.20	123.70 ± 49.23	4999.23 ± 5527.39	55.97 ± 50.48
After		5.51 ± 1.39 ^b^	72.88 ± 91.61 ^b^	55.83 ± 118.45 ^b^	0.26 ± 0.22	129.70 ± 52.45	4235.47 ± 4799.86 ^b^	32.19 ± 21.72 ^b^
**ALT Abnormal**	20							
Before		7.00 ± 1.80	113.75 ± 120.77	68.34 ± 70.49	0.25 ± 0.15	112.24 ± 41.13	7504.10 ± 7045.71	53.64 ± 87.24
After		6.23 ± 2.32	88.42 ± 99.14	33.43 ± 31.06	0.27 ± 0.20	137.49 ± 46.12	5257.85 ± 6445.95	35.29 ± 27.09

^b^ P < 0.05.

^a^Treg and Th17 cells frequencies are represented in percentage, the levels of cytokines are represented with pg/mL. Data are shown as mean ± standard deviation and analyzed by Wilcoxon signed-rank test. The frequencies of Treg cells, the levels of IL-10, TGF-β1 and IL-23 were significantly reduced when ALT normalized compared the values before the antiviral therapy.

4.4. Correlation Between Treg, Th17 Cells, HBV DNA, and ALT

**Figure 2.**
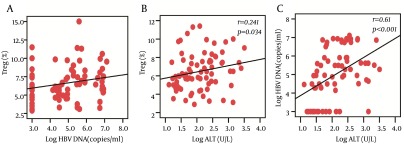
The Correlation Between Treg Cells Frequencies, HBV DNA Loads, and ALT Levels The Spearman's rank correlation was performed between the variables. The frequencies of Treg cells were positively correlated with HBV DNA load (A) and ALT levels (B). And the HBV DNA load was positively correlated with ALT levels (C).

We observed that the frequencies of Treg cells were positively correlated with HBV DNA load (r = 0.272, P = 0.016) and ALT levels (r = 0.241, P = 0.034) at baseline, while there was no significant correlation between Th17 frequency and HBV DNA load or ALT levels. In addition, we also found a significantly positive correlation between HBV DNA load and ALT levels (r = 0.61, P < 0.001) (Figure 2). 

## 5. Discussion

In the present study, the effects of NA on the virological and biochemical response in CHB patients were investigated. The results indicate that dynamic changes of Treg and Th17 cells and related cytokines closely correlate with the virological and biochemical responses in chronic hepatitis B patients undergoing NA treatment.

The immune response of host may affect the antiviral efficacy in CHB patients. The HBV-specific T-cell immune response is related to CD4 + T cell activity (23). CD4+T cell response was enhanced by lamivudine treatment within 1-2 weeks (24). Adefovir dipivoxil and entecavir combined therapy would also increase CD4 + T cell activity (25). Treg cells, as a subset of CD4 + T cell with immune suppression function, can inhibit HBV-specific T-cell immune response, thereby controlling liver inflammation and improving liver pathology, while it may not be conducive to HBV clearance (5, 6). Th17 cells are a newly discovered subset of CD4 + T cells, which are related to liver inflammation and fibrosis (10-12). But, the association between Th17 cells and antiviral efficacy has not been fully elucidated. In the present study, the results indicated that significantly decreased Treg cells and slightly increased Th17 cells were related to virological and biochemical responses.

Effectively, suppression of virus replication and successful reconstruction of T cell immune response in CHB patients are crucial for antiviral medications to obtain a lasting effect. Zhang JY et al. (13) reported that after entecavir treatment, the Treg cells frequencies decreased and Th17 cell frequencies increased, leading to a decline in Treg/Th17 ratio. Hence, the inhibitory ability of Treg cells to HBV-specific Th17 cells was weakened, and damaged Th17 cell function was partially restored, resulting in IL-17 secretion increase and antiviral enhancing. Zhang PY et al. (26) showed that after antivirus treatment, frequencies of Treg cells were decreased and Th17 increased, and immune response initiated mainly in the Th1 cells, contributing to viral replication suppression and virus clearance. Our experiment showed that in patients with complete or partial immune response, Treg cells frequencies were significantly decreased, and Th17 frequencies did not change significantly, resulting in a decline in Treg/Th17 ratio; for the non-immune response patients, frequencies of Treg cells and Th17 cells and levels of cytokines had no significant change. These results were similar to the above studies (13), and demonstrated that non-immune activator may also enhance the natural anti-HBV immunity. The possible reason is that inhibition of HBV replication by NA treatment causes cytokines environment changes, which on the one hand, leads to reduction of Treg cells frequencies and their inhibition function; and on the other hand, Th17 cells frequencies and IL-17 level increasing result in T-cell immune enhancement, and further break immune tolerance, which help to enhance the antiviral activity and clearing HBV. For patients with no response, it might be due to increased Treg cells inhibiting Th17 cells activity, leading to therapy failure.

Additionally, to better illustrate the association between Treg, Th17 cells and their associated cytokines and virological and biochemical responses, the patients were further divided into another 5 groups according to the degrees of HBV DNA decrease and normalization of ALT. We observed that the immune cells frequencies and related cytokines secretion changed significantly, when HBV DNA decreased markedly or ALT returned to normal. Especially, frequencies of Treg cells and levels of related cytokines IL-10 and TGF-β1 were significantly decreased. We also found that frequencies of Treg cells (but not Th17 cells) were positively correlated with HBV DNA load and ALT levels, which was similar to the previous study (27, 28). It means that the more active virus replication, the more Treg cell frequencies in patients. Active HBV replication may activate the Treg cells to suppress the immune response, thereby maintaining chronic HBV infection status, causing liver inflammation. After antiviral therapy, Treg cells frequencies were reduced and their immune suppression function to HBV-specific Th17 cells was weakened, thus further clearing the HBV, achieving virological and biochemical immune response. 

IL-23 is an important cytokine for Th17 cells survival, proliferation and functional maintenance (12). In the present study, 14.42 ± 13.08 weeks after NA therapy, IL-23 level was significantly lower than before, indicating that with duration of treatment, Th17 cells frequencies may also be gradually reduced, which thereby facilitates the liver inflammation. In our study, 24 weeks follow-up data (not included) confirmed that Th17 cells frequencies showed a descending tendency. Dynamically detection of Treg, Th17 cells and their related cytokines with more long-term follow-up may better expound their roles in the process of NA antiviral therapy. 

In summary, the present study demonstrated that in complete responders of chronic hepatitis B undergoing Nucleos(t)ide analogues antiviral therapy, frequencies of Treg cells and Th17 cells and levels of IL-10, TGF-β1, IL-23 and IL-17 were all changed compared to the values before the treatment, which is related to HBV DNA decline and ALT normalization. Detection of Treg cells and Th17 cells and their related cytokines has clinical significance in discovery of new therapeutic targets and evaluating the antiviral efficacy.
